# Convenient and Scalable Synthesis of Fmoc-Protected Peptide Nucleic Acid Backbone

**DOI:** 10.1155/2012/354549

**Published:** 2012-07-10

**Authors:** Trevor A. Feagin, Nirmal I. Shah, Jennifer M. Heemstra

**Affiliations:** Department of Chemistry and the Center for Cell & Genome Science, University of Utah, Salt Lake City, UT 84112, USA

## Abstract

The peptide nucleic acid backbone Fmoc-AEG-OBn has been synthesized via a scalable and cost-effective route. Ethylenediamine is mono-Boc protected, then alkylated with benzyl bromoacetate. The Boc group is removed and replaced with an Fmoc group. The synthesis was performed starting with 50 g of Boc anhydride to give 31 g of product in 32% overall yield. The Fmoc-protected PNA backbone is a key intermediate in the synthesis of nucleobase-modified PNA monomers. Thus, improved access to this molecule is anticipated to facilitate future investigations into the chemical properties and applications of nucleobase-modified PNA.

## 1. Introduction

**Figure 1:**
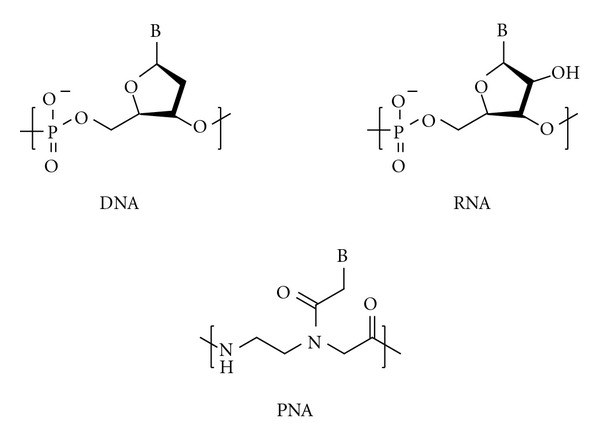
Chemical structure of DNA, RNA, and PNA.

**Figure 2:**
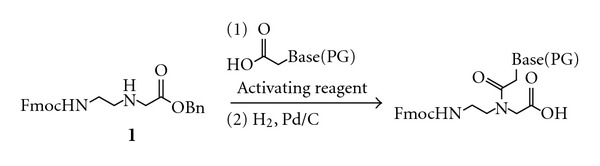
Synthesis of Fmoc-protected PNA monomers.

Peptide nucleic acid (PNA) [1] has recently emerged as a promising alternative to the native nucleic acids DNA and RNA (Figure 1) for a wide variety of applications including antisense therapy [2] and gene diagnostics [3]. The key advantages of PNA over DNA and RNA are its resistance to degradation by cellular nucleases [4] and its relatively higher binding affinity and mismatch selectivity in duplex formation [5]. PNA can be generated by Fmoc- or Boc-solid phase peptide synthesis [6], and Fmoc-protected monomers bearing each of the four canonical nucleobases are commercially available. Recently, the incorporation of modified nucleobases into PNA has been shown to enable synthesis of nucleic acids having unique physicochemical properties [7]. However, PNA monomers bearing modified nucleobases are not commercially available, and must instead be synthesized in the laboratory. Suitable reactions have been reported for preparation of modified nucleobases and coupling of these nucleobase acetic acids to the PNA backbone (Figure 2) [7–9]. However, to our knowledge, a scalable and cost-effective synthesis for the protected *N*-[2-(Fmoc)aminoethyl]glycine benzyl ester (Fmoc-AEG-OBn) backbone **1** has yet to be reported. Synthesis of the Fmoc-protected carboxylic acid backbone Fmoc-AEG-OH has been reported [10], and coupling of nucleobase acetic acids with Fmoc-AEG-OH has been described in the patent literature [11]. However, this coupling reaction provides moderate-to-low yields of PNA monomer [12, 13]. Here, we describe a synthesis of **1** that proceeds in four steps with an overall yield of 32%, utilizes inexpensive reagents, and can be scaled to produce large quantities of final product in a single batch with only minimal purification.

## 2. Materials and Methods

### 2.1. General Methods

Unless otherwise noted, all starting materials were obtained from commercial suppliers and were used without further purification. Flash column chromatography was carried out using silica gel 60 (230–400 mesh). ^1^H and ^13^C NMR chemical shifts are expressed in parts per million (*δ*) using residual solvent protons as internal standard (*δ* 7.26 ppm (^1^H) and 77.16 ppm (^13^C) for CHCl_3_). Coupling constants, *J*, are reported in Hertz (Hz), and splitting patterns are designated as s (singlet), d (doublet), t (triplet), q (quartet), m (multiplet), br (broad), and app (apparent). Mass spectra were obtained through the Mass Spectrometry Facility, University of Utah.

### 2.2. *tert*-Butyl(2-aminoethyl)carbamate (6)

A 2 L round bottom flask was charged with ethylenediamine (306.5 mL, 4.58 mol) and tetrahydrofuran (600 mL). Boc anhydride (50.0 g, 229 mmol) was dissolved in 400 mL tetrahydrofuran and added to the solution of ethylenediamine via addition funnel over 45 min with vigorous stirring. After 18 h, the reaction was quenched by addition of 500 mL H_2_O. The aqueous phase was saturated with solid K_2_CO_3_, then the phases were separated and the organic phase was dried over Na_2_SO_4_, filtered, and concentrated to give a pale yellow oil. The oil was dissolved in 700 mL toluene and concentrated to azeotrope remaining ethylenediamine, yielding 29.24 g of pale yellow oil (80%). ^1^H NMR (300 MHz, CDCl_3_) *δ* 4.90 (br s, 1H), 3.16 (q, *J* = 5.8 Hz, 2H), 2.78 (t, *J* = 5.9 Hz, 2H), 1.43 (s, 9H), 1.09 (br s, 2H). ^13^C NMR (75 MHz, CDCl_3_) *δ* 156.3, 79.1, 43.3, 41.8, 28.4. HRMS (ESI) *m/z* 161.1295 (calcd [M + H]^+^ = 161.1290).

### 2.3. Benzyl 2-((2-((tert-butoxycarbonyl)amino)ethyl)amino) acetate (9)

 A 2 L round bottom flask was charged with **6** (29.24 g, 183 mmol), triethylamine (25.5 mL, 183 mmol), and acetonitrile (450 mL). The reaction mixture was stirred and ethyl bromoacetate (28.9 mL, 182 mmol) added via syringe over 2 min. After 100 min, the reaction mixture was diluted with 500 mL EtOAc and washed with 500 mL 2 M K_2_CO_3_ (aq.), then 500 mL brine. The organic phase was dried over Na_2_SO_4_, filtered, and concentrated to a pale yellow oil, which was purified by flash column chromatography, (70 mm diameter column, 285 g silica gel, 1 : 1 hexanes : EtOAc, 99 : 1 EtOAc : Et_3_N) to give 40.56 g of pale yellow oil (72%). ^1^H NMR (300 MHz, CDCl_3_) *δ* 7.38–7.33 (m, 5H), 5.17 (s, 2H), 4.99 (br s, 1H), 3.45 (s, 2H), 3.20 (q, *J* = 5.7 Hz, 2H), 2.74 (t, *J* = 5.8 Hz, 2H), 1.59 (br s, 1H), 1.44 (s, 9H). ^13^C NMR (75 MHz, CDCl_3_) *δ* 172.3, 156.1, 135.5, 128.6, 128.4, 128.3, 79.0, 66.5, 50.4, 48.7, 40.1, 28.4. HRMS (ESI) *m/z* 331.1640 (calcd [M + Na]^+^ = 331.1634).

### 2.4. Benzyl 2-((2-((((9H-fluoren-9-yl)methoxy)carbonyl)amino)ethyl)amino)acetate (Fmoc-AEG-OBn 1)

To a 2 L round bottom flask was added **9** (40.46 g, 131 mmol) and dichloromethane (200 mL). The reaction mixture was stirred in an ice bath and 200 mL trifluoroacetic acid added. The ice bath was removed and the reaction mixture stirred for 20 min, then concentrated to a yellow oil. The oil was dissolved in 400 mL toluene and concentrated to azeotrope remaining trifluoroacetic acid. The resulting yellow oil was dissolved in 700 mL dichloromethane and stirred under N_2_ in an ice bath. Fmoc-OSu (44.26 g, 131 mmol) was added all at once, then triethylamine (54.8 mL, 393 mmol) was added dropwise via addition funnel over 5 min. The ice bath was removed and the reaction mixture was stirred for 2 h, then washed with 500 mL 1 M K_2_CO_3_ (aq.), dried over Na_2_SO_4_, filtered, and concentrated to a yellow oil. The oil was purified by flash column chromatography, (70 mm diameter column, 250 g silica gel, 1 : 1 hexanes : EtOAc, 98 : 2 EtOAc : MeOH) to give 31.04 g of pale yellow oil that crystallized into a white solid upon standing (55%). ^1^H NMR (300 MHz, CDCl_3_) *δ* 7.76 (d, *J* = 7.4 Hz, 2H), 7.61 (d, *J* = 7.3 Hz, 2H), 7.42–7.28 (m, 9H), 5.29 (br s, 1H), 5.18 (s, 2H), 4.40 (d, *J* = 7.0 Hz, 2H), 4.22 (t, *J* = 6.8 Hz, 1H), 3.47 (s, 2H), 3.29 (q, *J* = 5.4 Hz, 2H), 2.78 (t, *J* = 5.6 Hz, 2H), 1.58 (br s, 1H). ^13^C NMR (75 MHz, CDCl_3_) *δ* 172.3, 156.6, 144.0, 141.3, 135.5, 128.6, 128.5, 128.4, 127.6, 127.0, 125.1, 120.0, 66.6, 66.4, 50.4, 48.6, 47.3, 40.6. HRMS (ESI) *m/z* 431.1972 (calcd [M + H]^+^ = 431.1971).

## 3. Results

**Figure 3:**
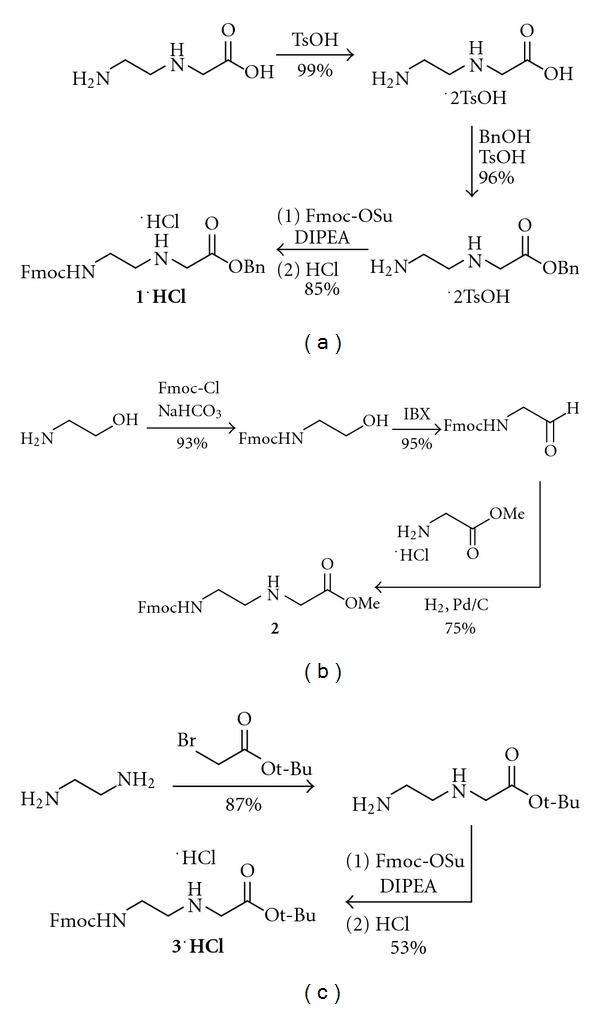
Reported synthetic routes to the Fmoc-AEG-OR backbone.

An extensive literature search reveals only one published route to **1** as shown in Figure 3(a). This route, reported by Hudson and coworkers, proceeds in three steps with an overall yield of 81%, but requires the use of costly *N*-(2-aminoethyl)glycine as the starting material [8]. Furthermore, in our hands, this synthetic route has proven difficult to reproduce. Alternatively, Porcheddu and coworkers have described a synthesis of Fmoc-AEG-OMe **2** that proceeds in three steps with an overall yield of 66% (Figure 3(b)), but requires three equivalents of IBX to accomplish the oxidation step [9]. Thomson and coworkers have described a synthesis of Fmoc-AEG-O*t*-Bu·HCl **3**·**HCl** that proceeds in two steps with 46% overall yield from inexpensive starting materials (Figure 3(c)), but does not produce analytically pure material [14]. Furthermore, the exocyclic amines of the PNA nucleobases are typically protected with acid-labile Boc or Bhoc protecting groups. Deprotection of the *tert*-butyl ester requires strongly acidic conditions, making **3** unsuitable for use with Boc- or Bhoc-protected nucleobases.

**Figure 4:**
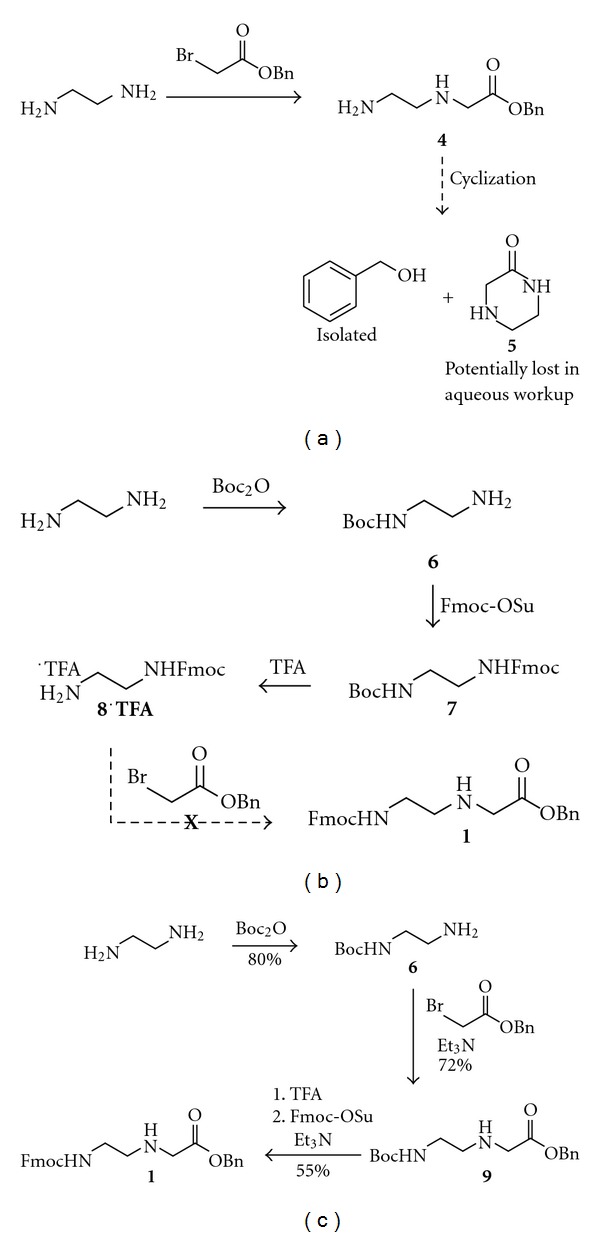
Synthetic route to Fmoc-AEG-OBn 1.

Inspired by the ease and cost-effectiveness of the Thomson route, we first envisioned synthesis of **1** by alkylation of ethylenediamine with benzyl bromoacetate, followed by reaction with Fmoc *N*-hydroxysuccinimide ester (Fmoc-OSu). Unfortunately, after alkylation and aqueous workup, only benzyl alcohol was recovered. We hypothesize that alkylation product **4** rapidly cyclizes to give benzyl alcohol and water soluble cyclic piperazinone **5** (Figure 4(a)), and that this undesired cyclization can only be suppressed by the use of a bulky ester such as a *tert*-butyl ester. In fact, this cyclization has been demonstrated previously using the analogous methyl ester [15]. We next considered reversing the order of the two reactions such that ethylenediamine would first be monoprotected with Fmoc-OSu, then alkylated with benzyl bromoacetate to give **1**. However, Fmoc-ethylenediamine cannot be directly prepared by the reaction of ethylenediamine with Fmoc-Cl or Fmoc-OSu. Rather, a three-step process is required in which ethylenediamine is mono-Boc protected (**6**), then Fmoc protected (**7**), and finally the Boc group is removed under acidic conditions to give **8** as the TFA salt [16]. Unfortunately, our attempts to alkylate **8**·**TFA** with benzyl bromoacetate failed to yield the desired product **1**, likely due to the instability of the free base of **8** (Figure 4(b)).

Fortunately, we were able to obtain Boc-ethylenediamine **6** in 80% yield from ethylenediamine and Boc anhydride using a modified version of a reported procedure [17], and this was successfully alkylated with benzyl bromoacetate to give **9** in 72% yield. We then deprotected the Boc group using trifluoroacetic acid (TFA) to give a quantitative yield of free amine, which was importantly found to be stable to cyclization when isolated as the TFA salt. In the final step, we combined the amine TFA salt with Fmoc-OSu prior to adding base, so that protection of the primary amine could compete with cyclization to give the desired product **1** in 55% yield. Starting with 50 g of Boc anhydride, we were able to generate 31 g of analytically pure **1** in a single batch using inexpensive reagents (Figure 4(c)) [18].

A key to the scalability of our synthetic route is the relatively facile purification of the synthetic intermediates and final product. The Boc protection step to give **6** requires only aqueous workup, and the deprotection step requires simple concentration and removal of TFA via formation of an azeotrope with toluene. The alkylation to produce **9** and the Fmoc protection to give **1** require flash column chromatography, but a large difference in *R*_*f*_ between the products and impurities makes purification possible using only a silica plug.

## 4. Discussion

Fmoc-protected PNA backbone **1** is a key intermediate in the synthesis of Fmoc-protected PNA monomers having modified nucleobases. However, to date, a scalable and cost-effective synthetic route to this molecule has yet to be reported in the literature. An efficient synthesis of the Boc-protected backbone has been reported, but our attempts to utilize this synthetic route with Fmoc in place of Boc failed to give product, likely due to the instability of synthetic intermediate **8**. Rather, synthesis of **1** can be initiated using a Boc protecting group, followed by a protecting group swap to provide the Fmoc-protected product. The first two steps of our synthetic route mirror those of the published synthesis for the Boc-protected monomer [19]. However, replacement of the Boc group with Fmoc poses a significant challenge, as this step proceeds through unstable intermediate **4**. We were able to perform this transformation by generating the free base of **4** at reduced temperature and in the presence of Fmoc-OSu, enabling Fmoc protection to effectively compete with cyclization, providing **1** in moderate yield.

In summary, we describe here a novel route to the PNA backbone Fmoc-AEG-OBn **1**. Using this route, we have rapidly synthesized 31 g of **1** using inexpensive starting materials and only minimal purification. The overall yield for our synthetic route is modest at 32%; however, the low cost of starting materials and ease of purification enable this synthesis to be tractable on a large scale. Having a convenient route to access **1** is anticipated to ease the synthesis of new Fmoc-protected PNA monomers, presumably furthering the exploration of PNA having unique modified nucleobases.
